# Improvements to dark experience replay and reservoir sampling for better balance between consolidation and plasticity

**DOI:** 10.3389/frai.2026.1649239

**Published:** 2026-02-19

**Authors:** Taisuke Kobayashi

**Affiliations:** National Institute of Informatics (NII), The Graduate University for Advanced Studies (SOKENDAI), Tokyo, Japan

**Keywords:** consolidation and plasticity, continual learning, dark experience replay, reinforcement learning, reservoir sampling

## Abstract

Continual learning is one of the most essential abilities for autonomous agents, which can incrementally learn daily-life skills even with limited computer resources. To achieve this goal, a simple yet powerful method called dark experience replay (DER) was recently proposed. DER mitigates catastrophic forgetting, where the skills acquired in the past are unintentionally forgotten when learning new skills, by stochastically storing streaming data in a reservoir sampling (RS) buffer and relearning them or retaining their past outputs. However, because DER considers multiple objectives, it does not function properly without appropriate weighting for each problem. In addition, the ability to retain past outputs inhibits learning if past outputs are inconsistent owing to distribution shifts or other effects. This is because of the trade-off between memory consolidation and plasticity. The trade-off is hidden even in the RS buffer, which gradually stops storing new data for new skills as data are continuously passed to it. To alleviate this trade-off and achieve a better balance, this study proposes improvement strategies for each DER and RS. Specifically, DER is improved by the automatic adaptation of weights, blocking of replaying inconsistent data, and correction of past outputs. RS is also improved with the generalization of acceptance probability, stratification of multiple buffers, and intentional omission of inconsistent data. These improvements were verified using multiple benchmarks including regression, classification, and reinforcement learning problems. Consequently, the proposed methods achieved a steady improvement in learning performance by balancing memory consolidation and plasticity.

## Introduction

Machine learning technologies have made remarkable progress in recent years (LeCun et al., 2015; Touvron et al., 2023; Kirillov et al., 2023), and the basic (self-)supervised learning framework typically relies on a large dataset prepared in advance. However, new data are constantly increasing over time, so new skills often arise in addition to those in the dataset used for training. Alternatively, owing to limited computational resources, it is not possible to have all huge datasets in memory or storage, inevitably missing several skills. The goal of continual learning (CL) (or lifelong learning) is to incrementally obtain new skills in a machine learning model, even in such situations (Parisi et al., 2019). In other words, CL must train the models using streaming data without a pre-prepared dataset and unlimited computational resources. Note that this is similar to reinforcement learning (RL) settings, but recent RL has improved performance by leveraging experience replay with a sufficiently large buffer, so RL systems with limited computational resources must handle CL.

In this CL problem setting, catastrophic forgetting (or catastrophic inference), in which previously obtained skills are forgotten when new skills are learned, is a major issue (McClelland et al., 1995). Therefore, the main objective of CL research is to alleviate this problem. Three major approaches have been proposed:

- Regularization: Among the parameters in the model (e.g., weights and biases of neural networks), the essential ones for representing past skills are selected (e.g., according to Fisher information), and then they are regularized to keep the current values (Kirkpatrick et al., 2017; Farajtabar et al., 2020). The remaining parameters are utilized to learn new skills. Alternatively, instead of regularizing the parameter space, regularization can be applied to the output space of the model to implicitly select and retain parameters important for representing past skills (Titsias et al., 2020; Khan and Swaroop, 2021).
- Rehearsal: By storing past data in a finite-size buffer, the model is trained not only on streaming but also on replayed data, enabling it to retain past skills even when acquiring new ones, similar to standard machine learning with a dataset (Chrysakis and Moens, 2020; Sun et al., 2022). Alternatively, instead of using a finite-size buffer, a data-generative model can be additionally trained to generate pseudo-past data, which can be used for learning (Shin et al., 2017; Pomponi et al., 2023).
- Modularization: Each skill is learned mainly in its corresponding module within a well-designed model, and the modules are prevented from learning other skills by restricting (especially, freezing) their updates (Kobayashi and Sugino, 2020; Kang et al., 2022). The number of representable skills can be increased by adding modules to the model as needed (Li et al., 2019; Ostapenko et al., 2021).

CL methods that combine these approaches have also been proposed (Buzzega et al., 2020; Daxberger et al., 2023). In addition to this categorization, CL methods can be classified according to whether each data has a label that explicitly represents the corresponding skill, task, or class (with or without information about when the target skill changes) (Aljundi et al., 2019b; Ye and Bors, 2022). Naturally, methods without label information are more general-purpose and realistic; however, it is well known that the difficulty of the problem increases significantly.

Among previous CL methods without label information, dark experience replay (DER) (more precisely, DER++ in the original study) has attracted attention as a simple yet powerful method (Buzzega et al., 2020). DER corresponds to a combination of rehearsal and regularization approaches. Specifically, DER stores past data and corresponding outputs from the model at that time in a buffer; and uses them to maintain the past skills and outputs in conjunction with learning new skills. Although DER has a simple implementation that does not require label information, it can significantly mitigate catastrophic forgetting.

However, behind the simple implementation, DER requires weighting for the simultaneous optimization of multiple objectives; therefore, it does not function properly without fine-tuning these weights for each problem. Indeed, while the original paper reported results on several benchmarks, DER used different weights across them. Furthermore, past outputs do not necessarily represent past skills (due to insufficient learning or distribution shifts); therefore, the model may attempt to preserve outputs that is inconsistent with the current situation. Additional regularization (Zhuo et al., 2023) and prioritized sampling (Wang et al., 2024) have been proposed as improvements to DER to reduce the impact of such errors, but they do not eliminate them and tend to make the learning process more conservative, thereby reducing plasticity, as described below.

The buffer used in DER is a reservoir sampling (RS) buffer (Vitter, 1985). While a first-in-first-out (FIFO) buffer, which is widely used in experience replay (Lin, 1992; Isele and Cosgun, 2018), always stores new data and discards the oldest data, the RS buffer stochastically stores new data and discards previously stored data. Thus, the RS buffer can be regarded as sampling a finite subset of data with uniform probability from among all the data seen so far, retaining a portion of the data corresponding to past skills. However, in other words, the older the data, the more opportunities they have to be included in the buffer, and the shorter the retention time for newer data. Although the buffer's utility can be increased by storing highly informative samples (Sun et al., 2022; Aljundi et al., 2019c; Brignac et al., 2023), this approach does not directly resolve this issue. Some approaches have been proposed to encourage the acceptance of new data by decaying the storage probability of older data (Cormode et al., 2009; Osborne et al., 2014), but excessive decay undermines the benefits of RS.

Thus, the conventional DER and RS methods used in this study have unresolved issues. In particular, perhaps because DER focuses on resolving catastrophic forgetting, it prioritizes the maintenance of past skills, resulting in a loss of plasticity for the efficient acquisition of new skills. This is related to a trade-off between consolidation^1^ and plasticity, which is a well-known problem faced by humans (Frank and Benington, 2006; Mermillod et al., 2013). Recently, it was reported that the plasticity of memory should be reconsidered (Dohare et al., 2024). Therefore, the current trend in which only consolidation is prioritized may be inappropriate. For example, as the distribution shift problem (Koh et al., 2021) suggests, past skills are not always correct and must be updated appropriately as the situation changes. Studies seeking a better balance between consolidation and plasticity often follow an approach that combines two models or structures that are biased in one direction or another (Jung et al., 2023; Kim et al., 2023), which increases the computational cost.

Therefore, this study seeks to improve DER and RS to achieve a better balance between memory consolidation and plasticity without introducing additional models or structures for satisfying limited computational resources. As a first contribution, a novel method called A2ER is proposed by incorporating three strategies into DER. Specifically, the *adaptation* strategy enables auto-tuning of the weights of DER and appropriately balances the learning from new data, learning from past data, and preservation of past outputs. Next, the *block* strategy suppresses the replay frequency of past data, which is inconsistent with the current models and data-generative distributions, for preventing the consolidation of wrong or unnecessary skills. Finally, the *correction* strategy corrects past outputs to make them consistent with the current situation and to increase plasticity.

As a second contribution, a new method called O2S is proposed by incorporating three strategies into RS. Specifically, the *q-logarithm* strategy generalizes the acceptance probability of the data passed to the RS buffer allowing for the specification of the balance between consolidation and plasticity. Next, the *plural* strategy prepares multiple RS buffers connected in series, which gradually shift from highly plastic to highly consolidated. Finally, the *omission* strategy deletes inconsistent past data when migrating data between buffers, leaving more important data in long-term memory.

These improvements are numerically verified using multiple benchmarks. First, to demonstrate the effectiveness of A2ER, classification and regression tasks on CL settings are solved with a small buffer, showing that A2ER yields higher accuracy than before owing to an appropriate learning balance and improved plasticity. Similarly, RL tasks are accomplished efficiently without consolidating inconsistent value functions or past policies. Next, to demonstrate the effectiveness of O2S, the underlying *q-logarithm* strategy is verified that it can specify a balance between consolidation and plasticity in a classification task involving a single distribution shift. Finally, O2S achieves higher generalization performance in goal-conditioned RL robotic tasks while reducing the amount of data passed to the RS buffers.

## Materials and methods

### Preliminaries

#### Continual learning

Let us consider the problem of CL, which is the subject of this study. First, an agent endlessly receives the input data $x_{t}\in X\subseteq {\mathbb{R}}^{\left|X\right|}$ (with *t* = 1, 2, … as the time step) from the environment it interacts with. The corresponding output data $y_{t}\in Y\subseteq {\mathbb{R}}^{\left|Y\right|}$ are predicted. *y*_*t*_ may be provided, as in supervised learning, or estimated by bootstrapping from *x*_*t*_ and other variables, as in RL; however, the former is assumed here for simplicity. Using a function approximator [e.g., deep neural networks (LeCun et al., 2015)] with parameters θ, the following minimization problem is solved:


$$\begin{array}{l} \theta^{*}=\arg\underset{\theta }{\min}\frac{1}{t}\overset{t}{\underset{\tau =1}{\sum }}L\left(g\left(z_{\tau }\right),y_{\tau }\right) \end{array}$$ (1)

$$\begin{array}{l} z_{\tau }=h_{\theta }\left(x_{\tau }\right) \end{array}$$

where $h_{\theta }:X\mapsto {\mathbb{R}}^{\left|Y\right|}$ denotes the function approximator to be optimized, and $g:{\mathbb{R}}^{\left|Y\right|}\mapsto Y$ denotes a fixed mapping function (e.g., a sigmoid function). By setting an appropriate loss function L, *y*≃*g*(*h*_θ_(*x*)) can be obtained.

The difficulty of continual learning stems from the fact that *t* continues to increase and its maximum cannot be defined. In extreme cases, when *t* → ∞, the aforementioned minimization problem cannot be numerically optimized. In addition, the data size of ${\left\{\left(x_{\tau },y_{\tau }\right)\right\}}_{\tau =1}^{t}$ is limited by finite computational resources, particularly for embodied systems such as robots. Therefore, the FIFO buffer $D^{\text{FIFO}}={\left\{\left(x_{\tau },y_{\tau }\right)\right\}}_{\tau =\min\left(0,t-N^{\text{FIFO}}\right)+1}^{t}$ of finite size *N*^FIFO^∈ℕ is often introduced, leading to the following surrogated minimization problem.


$$\begin{array}{l} \theta^{*}=\arg\underset{\theta }{\min}E_{D^{\text{FIFO}}}\left[L\left(g\left(h_{\theta }\left(x_{\tau }\right)\right),y_{\tau }\right)\right] \end{array}$$ (2)

This problem can be minimized using the stochastic gradient descent method (e.g., Ilboudo et al., 2023). In other words, a batch of data, the size of which is denoted by *B*, is randomly extracted from *D*^FIFO^, and θ is then updated using its gradient $\nabla_{\theta }|B{|}^{-1}\underset{\tau \in B}{\sum }L\left(g\left(h_{\theta }\left(x_{\tau }\right)\right),y_{\tau }\right)$. Although this solution enables optimization as in general deep learning, it discards past data from the optimization when *t*>*N*^FIFO^. As *t* increases, the skills acquired from τ ≤ min(0, *t*−*N*^FIFO^) are overwritten (unless the data in the FIFO buffer contain equivalent skills). This type of overwriting is known as catastrophic forgetting (McClelland et al., 1995).

#### Dark experience replay

Several approaches have been proposed to mitigate catastrophic forgetting. Among them, DER (Buzzega et al., 2020) (more precisely, DER++ in the original paper) is employed as a baseline of this study with slight modifications. DER is a simple yet powerful continual learning method that can be regarded as a combination of rehearsal and functional regularization. DER introduces the RS buffer (Vitter, 1985), *D*^RS^ (with size *N*^RS^), to store past data that i) is passed in streaming format (the original implementation); or ii) overflow from the FIFO buffer (this paper's implementation). This RS buffer stochastically stores all the data seen so far with equal probability (see the next section), rather than storing the latest data first as in the FIFO buffer. In addition, feature *z*_*t*_ computed using *h*_θ_ is stored in the RS buffer together with (*x*_*t*_, *y*_*t*_).

Under this design, the following minimization problem is solved:


$$\begin{array}{l} \theta^{*}=\arg\underset{\theta }{\min}\left(1-\beta \right)E_{D^{\text{FIFO}}}\left[L\left(g\left(h_{\theta }\left(x_{\tau }\right)\right),y_{\tau }\right)\right]\\ +\beta E_{D^{\text{RS}}}\left[L\left(g\left(h_{\theta }\left(x_{\tau }\right)\right),y_{\tau }\right)\right] \end{array}$$

$$\begin{array}{l} +\alpha E_{D^{\text{RS}}}\left[\frac{1}{2}||h_{\theta }\left(x_{\tau }\right)-z_{\tau }|{|}_{2}^{2}\right] \end{array}$$ (3)

where β∈[0, 1] is the coefficient that adjusts the learning priority between the FIFO and RS buffers, and α≥0 is the weight of the regularization term that preserve past features computed using the previous θ. The second and third terms randomly select batches from *D*^RS^. In the original implementation, each batch was selected independently; in this study, however, all data were selected simultaneously and split into two non-overlapping batches to strengthen the regularization of DER. This implementation can be regarded as a generalized version of CLEAR (Rolnick et al., 2019), which was originally designed for RL.

#### Reservoir sampling

As mentioned previously, the RS buffer used in DER stochastically stores all the data seen so far with equal probability (Vitter, 1985). Once the buffer is full, the following algorithm is applied to select the discarded data *d*^del^ based on the new data arriving *n*-th data point, *d*′, and the existing entries ${\left\{d_{i}\right\}}_{i=1}^{N^{\text{RS}}}$.


$$\begin{array}{l} \;k\sim U(1,n)\;\\ d^{\text{del}}=\{\begin{array}{ll} d^{\prime } & k>N^{\text{RS}}\\ d_{k} & 1\leq k\leq N^{\text{RS}} \end{array} \end{array}$$ (4)

where U(l,u) denotes a discrete uniform distribution over integers in the interval [*l, u*], *l, u*∈ℤ and *l* ≤ *u*. When data are discarded from the buffer, the new data replace the corresponding index as $d_{k}=d^{\prime }$.

In the above algorithm, the probability of accepting the new data *d*_*n*_ on the *n*-th pass is given as follows:


$$\begin{array}{l} P\left(d_{n}\in D_{n}^{\text{RS}}\right)\\ =P\left(1\leq k\leq N^{\text{RS}};k\sim U\left(1,n\right)\right) \end{array}$$

$$\begin{array}{l} =\overset{N^{\text{RS}}}{\underset{k=1}{\sum }}\frac{1}{n}=\frac{N^{\text{RS}}}{n} \end{array}$$ (5)

where *P*(·) denotes the probability of satisfying the condition in parentheses. In addition, the probability that it remains in the RS buffer after *n*′ = 1, 2, … additional steps is given by


$$\begin{array}{l} P\left(d_{n}\in D_{n+n^{\prime }}^{\text{RS}}\right)\\ =P\left(d_{n}\in D_{n+n^{\prime }-1}^{\text{RS}}\right)P\left(d_{n}\neq d_{n+n^{\prime }}^{\text{del}}\right)\\ =P\left(d_{n}\in D_{n+n^{\prime }-1}^{\text{RS}}\right)\left(\frac{n+n^{\prime }-N^{\text{RS}}}{n+n^{\prime }}+\frac{N^{\text{RS}}}{n+n^{\prime }}\frac{N^{\text{RS}}-1}{N^{\text{RS}}}\right)\\ =P\left(d_{n}\in D_{n}^{\text{RS}}\right)\overset{n^{\prime }}{\underset{m=1}{\prod }}\frac{n+n^{\prime }-m}{n+n^{\prime }+1-m} \end{array}$$

$$\begin{array}{l} =\frac{N^{\text{RS}}}{n+n^{\prime }} \end{array}$$ (6)

These two probabilities show that the RS buffer stochastically stores all data seen so far with equal probability, inversely proportional to the total number of data points passed, *n*. Here, *n* is sometimes referred to as the reservoir counter (Sun et al., 2022).

#### Related work

In previous research on continual learning, various improvements have been proposed for rehearsal-based approaches, which involve deep engagement with DER and RS. For example, many methods (Buzzega et al., 2021; Boschini et al., 2022; Caccia et al., 2022; Harun et al., 2025) utilize class (or task) information to adjust the balance between data to be rehearsed and stored, or to redefine the loss function. However, such approaches are inappropriate for this study, which assumes a task-agnostic setting.

Without using task information, determining data value often requires significant additional computational cost. For example, Aljundi et al. (2019c),(a) evaluate high-importance data using gradient information or loss changes before and after updates. In either case, gradients must be computed separately from parameter learning, doubling computational cost. Before adding data to RS buffer, Sun et al. (2022) judges a new metric akin to information gain based on data novelty and learnability. However, similarly to the above, this computation also requires substantial redundant computation and models. Bayesian neural networks need to be introduced for estimating data uncertainty, another metric for data importance, taking high cost (Wang et al., 2024). Thus, while several metrics for selecting data to replay and store have been proposed, all incur substantial computational costs solely for value estimation, making them unsuitable for systems with limited computational resources.

Another development involves revising the regularization terms introduced in DER and proposing new regularization terms. For example, Zhuo et al. (2023) proposes the forward consistency loss, although its computation requires retaining past model parameters. In the recent theoretical work (Daxberger et al., 2023), a loss integrating rehearsal, function regularization, and parameter-space regularization has been derived. However, its implementation still requires additional weight adjustment and estimation for parameter-space regularization compared to DER. Thus, regularization terms are not easily added, and in practice, they increase fine-tuning effort. This makes them difficult to handle in this study, which assumes scenarios lacking domain knowledge.

From the above, it is evident that under the task-agnostic setting with limited computational resources as the premise assumed by this study, the development of DER and related methods is not well-suited. Therefore, the subsequent discussion will proceed with the standard DER as the baseline, constructing a methodology that satisfies the aforementioned premise while enabling flexible adjustment of the trade-off between memory consolidation and plasticity.

### Improvements of DER: A2ER

#### Open issues in DER

DER is an effective method that can strongly mitigate catastrophic forgetting despite its simple implementation. However, the hyperparameters β and α in DER must be appropriately tuned, depending on the target problem, to achieve optimal performance. In fact, the original study (Buzzega et al., 2020) used different values for each benchmark. In other words, the balance between memory consolidation and plasticity must be determined manually and often requires considerable effort.

Additionally, the third term in Equation 3, which corresponds to functional regularization, attempts to preserve past features, even if they are inconsistent with the current situation. That is, the features may be valid only for past situations and not after distribution shifts. In that case, plasticity is more preferable than consolidation by the regularization.

**Figure 1:**
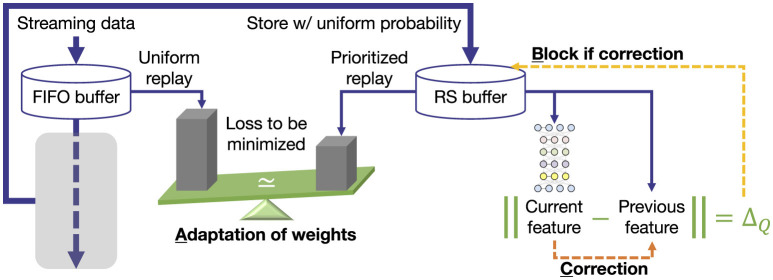
A2ER with three strategies for enhancing DER.

To address these issues, this study proposes A2ER, which incorporates three strategies into DER (see Figure 1): *adaptation, block*, and *correction*.

#### Adaptation of weights

Here, the *adaptation* strategy aims to automatically adjust β and α in DER, inspired by recent machine learning formulations (Haarnoja et al., 2018; Stooke et al., 2020). Specifically, the minimization problem in Equation 3 can be reinterpreted as the minimization of Equation 2 subject to the following equality constraints, which correspond to the second and third terms in DER:


$$\begin{array}{l} E_{D^{\text{RS}}}\left[L\left(g\left(h_{\theta }\left(x_{\tau }\right)\right),y_{\tau }\right)\right]=E_{D^{\text{FIFO}}}\left[L\left(g\left(h_{\theta }\left(x_{\tau }\right)\right),y_{\tau }\right)\right] \end{array}$$ (7)

$$\begin{array}{l} E_{D^{\text{RS}}}\left[\Delta_{\tau }\right]=\Delta_{Q} \end{array}$$ (8)

where $\Delta_{\tau }=||h_{\theta }\left(x_{\tau }\right)-z_{\tau }|{|}_{2}^{2}/2$ for brevity, and Δ_*Q*_≥0 denotes a threshold value for the average of Δ_τ_.

In this study, Δ_*Q*_ is heuristically defined as a variable updated at each step using the following quantile function *Q*:


$$\begin{array}{l} \Delta_{Q}=\left(1-\frac{\left|B\right|}{N^{\text{RS}}}\right)\Delta_{Q}+\frac{\left|B\right|}{N^{\text{RS}}}Q\left({\left\{\Delta_{\tau }\right\}}_{\tau \in B};\rho \right) \end{array}$$ (9)

where ρ∈(0, 1) denotes the quantile level.

These equality constraints can be converted into regularization terms using Lagrange multipliers with β and α. By considering Δ_*Q*_ as a constant that is independent of θ, this conversion is consistent with Equation 3. Furthermore, β and α can be optimized to satisfy the equality constraints, yielding the following auto-tuning rules:


$$\beta^{*}=\arg\underset{\beta }{\min}-\beta \{E_{D^{\text{RS}}}[ℒ(g(h_{\theta }(x_{\tau })),y_{\tau })]$$

$$-E_{D^{\text{FIFO}}}[ℒ(g(h_{\theta }(x_{\tau })),y_{\tau })]\}$$ (10)

$$\alpha^{*}=\arg\underset{\alpha }{\min}-\alpha \left\{E_{D^{\text{RS}}}\left[\Delta_{\tau }\right]-\Delta_{Q}\right\}$$ (11)

These are also solved by stochastic gradient descent, together with the minimization problem in Equation 3. Note that although the Lagrange multipliers (i.e., β and α in this case) are originally real numbers, their respective domains are restricted to β∈[0, 1] and α≥0. These constraints can be enforced using sigmoid and softplus functions, respectively. Even with these transformations, β and α can be efficiently optimized using the mirror descent method (Beck and Teboulle, 2003).

With the above formulation, β related to the first constraint brings about a proper balance between consolidation and plasticity by ensuring that the latest data in the FIFO buffer and the past data in the RS buffer incur similar levels of loss. The second constraint, governed by α, empirically strengthens consolidation by suppressing excessive changes in features, and increases plasticity by reducing functional regularization after sufficient consolidation. Although ρ is added for computing the threshold Δ_*Q*_, its tuning is task-agnostic and robust (see the experimental results later).

#### Block of replays and correction of features

In the second equality constraint, past data that exceed Δ_*Q*_ are classified as either inconsistent owing to distribution shifts or as having non-optimized features owing to insufficient learning. Therefore, the *block* strategy restricts the replay probability of such inconsistent data with the current situation, similar to intentional forgetting (Johnson, 1994), whereas the *correction* strategy updates the features in the buffer if the error is caused by a lack of learning similar to memory engram updating (Josselyn and Tonegawa, 2020). Because these strategies share many underlying processes, they are introduced together in this section.

First, it is assumed that the more Δ_τ_ deviates from Δ_*Q*_, the more the features are considered inconsistent (or non-optimized). Therefore, the following quantity η_τ_∈[0, 1] is designed to be proportional to the degree of deviation.


$$\begin{array}{l} \eta_{\tau }=1-\frac{\text{clip}\left(\Delta_{\tau };\Delta_{Q},{\bar{\Delta }}_{Q}\right)-\Delta_{Q}}{{\bar{\Delta }}_{Q}-\Delta_{Q}} \end{array}$$ (12)

$$\begin{array}{l} {\bar{\Delta }}_{Q}=\frac{1}{\rho }\Delta_{Q} \end{array}$$ (13)

where clip(*x*; *l, u*) is a function that clips *x* to the interval [*l, u*], where *l, u*∈ℝ and *l* ≤ *u*. ${\bar{\Delta }}_{Q}$ serves as an approximate empirical estimate of the maximum Δ_τ_.

Using the calculated η_τ_, the following update of Δ_τ_ is considered (by correcting *z*_τ_):


$$\begin{array}{l} \Delta_{\tau }\leftarrow \eta_{\tau }\Delta_{\tau }+\left(1-\eta_{\tau }\right)\Delta_{Q} \end{array}$$ (14)

**Figure 2:**
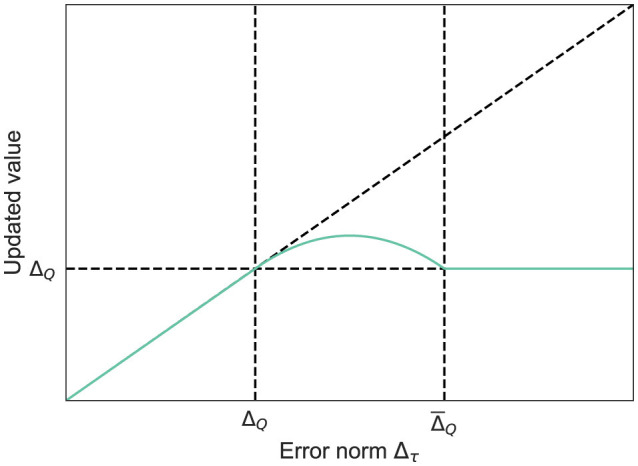
Updated Δ_τ_ using η_τ_.

With this design, Δ_τ_ in the range $\Delta_{Q}\leq \Delta_{\tau }\leq {\bar{\Delta }}_{Q}$ forms a quadratic curve with a maximum at η_τ_ = 1/2, and returns to Δ_*Q*_ at η_τ_ = 0, 1, as illustrated in Figure 2. That is, up to the midpoint, Δ_τ_ is expected to be minimized through the auto-tuning of α; after that, the behavior shifts to the correction of *z*_τ_.

The correction rate γ_τ_∈[0, 1] of *z*_τ_ toward *h*_θ_(*x*_τ_) (i.e., the current features) is defined as follows:


$$\begin{array}{l} \eta_{\tau }\Delta_{\tau }+\left(1-\eta_{\tau }\right)\Delta_{Q}\\ =\frac{1}{2}||h_{\theta }\left(x_{\tau }\right)-\left(1-\gamma_{\tau }\right)z_{\tau }-\gamma_{\tau }h_{\theta }\left(x_{\tau }\right)|{|}_{2}^{2}\\ =\frac{1}{2}||\left(1-\gamma_{\tau }\right)\left\{h_{\theta }\left(x_{\tau }\right)-z_{\tau }\right\}|{|}_{2}^{2}\\ ={\left(1-\gamma_{\tau }\right)}^{2}\Delta_{\tau } \end{array}$$

$$\begin{array}{ll} ∴ & \gamma_{\tau }=1-{\left\{\frac{\eta_{\tau }\Delta_{\tau }+\left(1-\eta_{\tau }\right)\Delta_{Q}}{\Delta_{\tau }}\right\}}^{\frac{1}{2}} \end{array}$$ (15)

The larger γ_τ_ is, the more inconsistent past data are. The *block* strategy suppresses the use of past data themselves for replay and learning. Specifically, although the expectation over *D*^RS^ in Equation 3 normally replays all data with uniform probability $p_{\tau }=1/N^{\text{RS}}$, each probability is instead weighted as follows:


$$\begin{array}{l} p_{\tau }=\frac{{\bar{\gamma }}_{\tau }}{\underset{\tau^{\prime }}{\sum }{\bar{\gamma }}_{\tau^{\prime }}} \end{array}$$ (16)

$$\begin{array}{ll} {\bar{\gamma }}_{\tau } & \leftarrow \left(1-\lambda \right){\bar{\gamma }}_{\tau }+\lambda \left(1-\gamma_{\tau }\right) \end{array}$$

where λ∈(0, 1) denotes the hyperparameter for the exponential moving average of 1−γ_τ_. That is, if the error norm does not become sufficiently small even after repeated learning and correction, the data will be gradually blocked for replay. Note that λ would be of low importance because it works well even when set relatively large (i.e., slight smoothing effect).

In the *correction* strategy *z*_τ_ in the buffer is updated using γ_τ_ as follows:


$$\begin{array}{l} z_{\tau }\leftarrow \left(1-\gamma_{\tau }\right)z_{\tau }+\gamma_{\tau }h_{\theta }\left(x_{\tau }\right) \end{array}$$ (17)

The minimization problem in Equation 3 can be solved after this correction. In practice, this can be done by appropriately compensating the loss function associated with *z*_τ_. That is, Δ_τ_−Δ_*Q*_ in the update rule for α in Equation 11 is multiplied by η_τ_, and Δ_τ_ in the update rule for θ in Equation 3 is multiplied by ${\left(1-\gamma_{\tau }\right)}^{2}$. In this way, additional computations can be avoided by reusing values already stored in RAM.

### Improvements of RS: O2S

#### Open issues in RS

The RS buffer used in DER accepts and stores data with a probability inversely proportional to the reservoir counter *n*, as expressed in Equations 5 and 6. In other words, when *n* becomes very large, new data are rarely accepted into the RS buffer; however, if accepted once, they can be stored for a long time. This characteristic is effective in preventing past data from being discarded and in improving consolidation, while it impairs plasticity needed to adapt to distributional shifts.

In addition, data have varying degrees of importance, as suggested by the *block* strategy, and it is undesirable to pass inconsistent data to the RS buffer, as noted in previous work (Sun et al., 2022). In other words, if inconsistent data are stored in the RS buffer, they may impede learning. Furthermore, simply passing such data to the RS buffer increases *n*, which reduces the acceptance rate for more important data that may arrive later.

**Figure 3:**
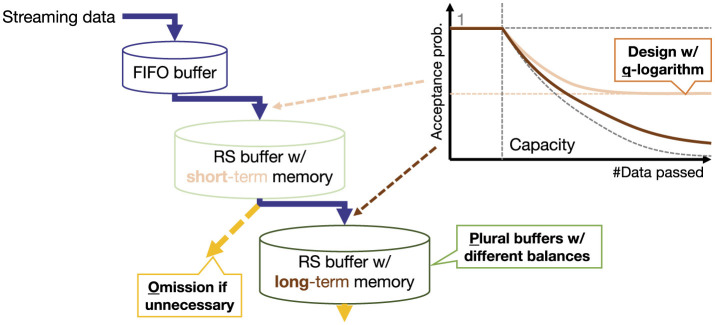
O2S with three strategies for improving RS.

To resolve these issues, this study proposes O2S, which introduces three strategies to enhance RS (see Figure 3), *q-logarithm, plural*, and *omissions*.

#### Generalization of acceptance rate using q-logarithm

First, the acceptance rate is generalized to make a balance between consolidation and plasticity adjustable. Specifically, a monotonically nondecreasing transformation *f*:ℤ↦ℤ is designed to generate random numbers for *n*>*N*^RS^, such that sampling in Equation 4 becomes k~U(1,f(n)). Note that *f* is required to be monotonically nondecreasing because it is also interpreted as a counter. The acceptance rate of the *n*-th new data point *d*_*n*_ is given as follows:


$$\begin{array}{l} P\left(d_{n}\in D_{n}^{\text{RS}}\right)=\frac{N^{\text{RS}}}{f\left(n\right)} \end{array}$$ (18)

To satisfy the definition of probability and maintain continuity with the case *n* ≤ *N*^RS^, where the acceptance rate is 1, it must hold that $\underset{n\to N^{\text{RS}}}{\lim}f\left(n\right)=N^{\text{RS}}$.

Considering the probability that *d*_*n*_ remains in the RS buffer at time *n*+*n*′-th, one term cannot be canceled out, although the derivation procedure is similar to Equation 6.


$$\begin{array}{l} P\left(d_{n}\in D_{n+n^{\prime }}^{\text{RS}}\right)\\ =P\left(d_{n}\in D_{n+n^{\prime }-1}^{\text{RS}}\right)P\left(d_{n}\neq d_{n+n^{\prime }}^{\text{del}}\right)\\ =P\left(d_{n}\in D_{n+n^{\prime }-1}^{\text{RS}}\right)\\ \;\times \left(\frac{f\left(n+n^{\prime }\right)-N^{\text{RS}}}{f\left(n+n^{\prime }\right)}+\frac{N^{\text{RS}}}{f\left(n+n^{\prime }\right)}\frac{N^{\text{RS}}-1}{N^{\text{RS}}}\right)\\ =P\left(d_{n}\in D_{n+n^{\prime }-1}^{\text{RS}}\right)\left(\frac{f\left(n+n^{\prime }\right)-1}{f\left(n+n^{\prime }\right)}\right) \end{array}$$

$$\begin{array}{l} =\frac{N^{\text{RS}}}{f\left(n+n^{\prime }\right)}\overset{n^{\prime }}{\underset{m=1}{\prod }}\frac{f\left(n+m\right)-1}{f\left(n+m-1\right)} \end{array}$$ (19)

Here, the first term corresponds to the conventional case with *f*(*n*) = *n*, whereas the second term—that is, the total product operation—modifies it.

Even with such modifications, the definition of probability must still be satisfied. To this end, the condition for designing *f* can be derived by focusing on the inner part of the second term.


$$\begin{array}{l} \frac{f\left(n+m\right)-1}{f\left(n+m-1\right)}=\frac{\Delta f\left(n+m\right)+f\left(n+m-1\right)-1}{f\left(n+m-1\right)} \end{array}$$

$$\begin{array}{l} =1+\frac{\Delta f\left(n+m\right)-1}{f\left(n+m-1\right)} \end{array}$$ (20)

where Δ*f*(*n*) = *f*(*n*)−*f*(*n*−1). If this term ever exceeds 1, then the resulting probability may also exceed 1, violating the definition of probability. Therefore, the sufficient condition Δ*f*(*n*) ≤ 1 must be satisfied.

Thus, the generalized counter *f*(*n*) must satisfy the following conditions:


$$\begin{array}{l} \begin{array}{c} \underset{n\to N^{\text{RS}}}{\lim}f\left(n\right)=N^{\text{RS}}\\ 0\leq \Delta f\left(n\right)\leq 1 \end{array} \end{array}$$ (21)

When Δ*f*(*n*) = 1, new and past data are equally likely to be included in the buffer after each update, promoting the storage of past data and supporting consolidation. When Δ*f*(*n*) = 0, the probability of past data remaining in the buffer decays exponentially because of the cumulative product, as in the case where *f*(*n*) = *c* at convergence. This facilitates the acceptance of new data and enhances plasticity. In other words, if Δ*f*(*n*) can be specified and adjusted to a suitable degree, it becomes possible to balance between consolidation and plasticity.

There are several possible candidates for functions that satisfy these conditions, but this study introduces the following *q*-logarithmic function (Tsallis, 1988), referred to as the *q-logarithm* strategy:


$$\begin{array}{l} f_{q}(n)=\min(n,N^{\text{RS}})\\ \;+\left\lfloor N^{\text{RS}}\ln_{q}\left(1+\frac{\max(0,n-N^{\text{RS}})}{N^{\text{RS}}}\right)\right\rfloor \\ \ln_{q}(x)=\{\begin{array}{ll} \ln(x) & q=1\\ \frac{x^{1-q}-1}{1-q} & q\neq 1 \end{array} \end{array}$$ (22)

where *q*∈[0, 2] is a hyperparameter that balances consolidation and plasticity.

**Figure 4:**
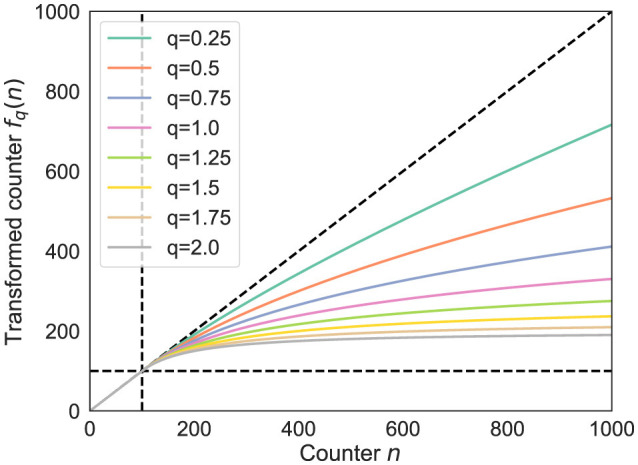
Example of *f*_*q*_(*n*) with *N*^RS^ = 100.

*f*_*q*_(*n*) is shown in Figure 4. As shown in the figure, for *q*_1_>*q*_2_, *f*_*q*_1__(*n*) ≤ *f*_*q*_2__(*n*) holds. For *q* = 0, *f*_*q*_(*n*) reduces to the conventional RS with *f*_*q* = 0_(*n*) = *n*, which yields the highest consolidation. For 0 ≤ *q* ≤ 1, $\underset{n\to \infty }{\lim}f_{q}\left(n\right)=\infty$, meaning the buffer eventually stops accepting new data and storing past data, although there is a time lag before convergence. On the other hand, for *q*>1, an upper bound exists, and the function converges to $\underset{n\to \infty }{\lim}f_{q}\left(n\right)=N^{\text{RS}}+N^{\text{RS}}/\left(q-1\right)$. Therefore, new data are accepted finally with constant probability and past data decay exponentially, increasing plasticity. Note that although *q* → ∞ is theoretically valid, the acceptance rate of new data converges to 0.5 with *q* = 2, which offers sufficient plasticity. This study therefore restricts *q* to the interval [0, 2], as previously defined.

Thus, a small *q* increases consolidation and a large *q* increases plasticity, allowing continuous adjustment of the balance between them. Other possible simple designs are examined in Appendix 4.

#### Plural buffers and omission of data passing

Although the designed counter *f*_*q*_ can adjust the balance between consolidation and plasticity, the trade-off itself is not eliminated and may lead to suboptimal performance unless it is appropriately tuned for the target problem. To alleviate this limitation, the *plural* strategy introduces a layered structure with multiple RS buffers configured differently, similar to human short- and long-term memory systems (Cowan, 2008). Furthermore, when passing data between multiple RS buffers, the importance of each data point is calculated in terms of sampling priority. Therefore, the *omission* strategy uses this information to determine whether the data should be passed to the next buffer.

**Figure 5:**
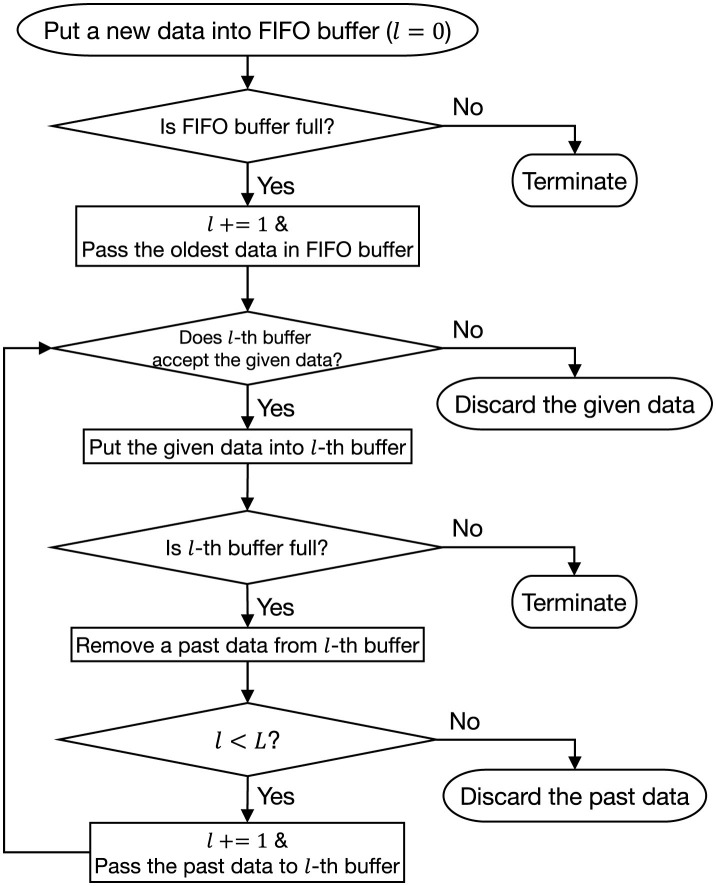
Data processing in *plural* strategy with *L* RS buffers.

First, the *plural* strategy prepares *L*∈ℕ layers of serially connected RS buffers ${\left\{D_{l}^{\text{RS}}\right\}}_{l=1}^{L}$ with respective sizes $N_{l}^{\text{RS}}$. Each has its own counter *n*_*l*_ and balance *q*_*l*_ for *f*_*q*_ in Equation 22. Here, the FIFO buffer is regarded as a special buffer at *l* = 0. Data processing is shown in Figure 5. When the past data in the *l*−1-th buffer are discarded, they are passed to the *l*-th buffer. When new data passed to the *l*−1-th buffer are not accepted, they are not passed to the *l*-th buffer. If *q*_*l*_ is small and consolidation is prioritized in the shallow layers of *l*≃1, the buffer rarely discards past data, thereby preventing the flow to subsequent layers. Therefore, for *l*_1_<*l*_2_, it is desirable that *q*_*l*_1__≥*q*_*l*_2__. In this way, buffers in deeper layers of *l*≃*L* will have a longer time scale because new data will be passed to them less frequently. Note that the batch for training in Equation 3 is constructed as the sum of sub-batches (each of size |*B*|/*L*) sampled from all RS buffers.

Second, the *omission* strategy intercepts the data passing from the *l*−1-th to the *l*-th buffers. As previously mentioned, each data point has an importance value represented by the replay priority $\bar{\gamma }$. In other words, data that have already been, or will be, blocked from replay tend to waste buffer capacity; therefore, the buffer should be fully utilized by discarding this data before advancing its counter or passing it to the next buffer. By converting $\bar{\gamma }$ into rejection probabilities for each buffer $p_{l-1,l}^{\text{rej}}$, a data point at time τ is passed to the *l*-th buffer as $d_{l}^{\prime }$ with the following rejection probability *p*^rej^:


$$\begin{array}{l} p^{\text{rej}}=1-\left(1-p_{l-1}^{\text{rej}}\right)\left(1-p_{l}^{\text{rej}}\right) \end{array}$$ (23)

$$\begin{array}{l} p_{l-1,l}^{\text{rej}}={\left(\frac{{\bar{\gamma }}_{l-1,l}^{\text{max}}-{\bar{\gamma }}_{\tau }}{{\bar{\gamma }}_{l-1,l}^{\text{max}}-{\bar{\gamma }}_{l-1,l}^{\text{min}}}\right)}^{\nu } \end{array}$$

where ${\bar{\gamma }}_{l-1,l}^{\text{max}}$ and ${\bar{\gamma }}_{l-1,l}^{\text{min}}$ denote the maximum and minimum values in either the *l*−1- or *l*-th buffer, and ${\bar{\gamma }}_{\tau }$ is the replay priority for the current data. ν≥0 adjusts the rejection rate; however, it is difficult to tune intuitively. Instead, this paper specifies the rejection probability ζ∈[0, 1], setting $p_{l-1}^{\text{rej}}=p_{l}^{\text{rej}}=0.5^{\nu }$ (for data with intermediate priority), which yields $\nu =-\ln\left(1-\sqrt{1-\zeta }\right)/\ln\;2$. Note that because the FIFO buffer does not have $\bar{\gamma }$, the data passed from *l* = 0 to *l* = 1 are always accepted, with $p_{0}^{\text{rej}}=0$. In addition, for numerical stability, $p_{\cdot }^{\text{rej}}=0$ if ${\bar{\gamma }}_{\cdot }^{\text{max}}-{\bar{\gamma }}_{\cdot }^{\text{min}}$ is less than ϵ (in this study, 10^−5^).

## Results

### Common setup

Multiple numerical benchmarks were used to validate the effectiveness of the proposed A2ER and O2S methods, the pseudo-codes of which are summarized in Appendix 1. The basic hyperparameter settings were the same across all benchmarks to confirm the task-agnostic design. Specifically, following the settings provided in DER (Buzzega et al., 2020), the FIFO buffer size, *N*^FIFO^, was set to 512, and the RS buffer size, *N*^RS^, was also 512 (in total, even when the *plural* strategy was applied). The batch size, the number of data replayed from each buffer at once, was set to |*B*| = 32. The initial value of α in Equation 3 was set to 1, and the initial value of β was 0.5. These are the most natural values in the absence of prior knowledge for problem, and they should also be used in DER as well to illustrate that DER without fine-tuning degrades its performance. The training frequency relative to the incoming data, *H*, and the number of batches replayed per training step, *E*, were specified individually for each benchmark. Additionally, AdaTerm (Ilboudo et al., 2023) was employed by default for optimization using stochastic gradient descent. Note that the computational cost of this setting is summarized in Appendix 2.

Among the hyperparameters involved in the proposed method, ρ in Equation 9, which defines the quantile function, appears to be particularly important, and its influence was investigated in Appendix 3. Consequently, ρ = 0.5 (i.e., the median value) was found to be robustly appropriate. Although there is room for adjusting λ, defined in Equation 17, it was set to 0.5, which is the midpoint of its domain, for simplicity. Note that since λ = 0.5 does not provide much smoothing, it might be allowable not to introduce ${\bar{\gamma }}_{\tau }$. *q* required for Equation 17 was set to 1 for the last buffer, based on comparisons with other designs presented in Appendix 4. For simplicity, *L* = 2 was used for the *plural* strategy in this study, and for the first buffer, *q* = 1.5 was used to emphasize plasticity. Finally, the probability of rejecting inconsistent data when transferring between buffers was empirically set to ζ = 0.2, so that excessive counter growth could be suppressed to some extent via rejection.

The above settings are summarized in Table 1. Although there is room for fine-tuning these settings depending on the problem, this does not appear to be very important because the effectiveness of the proposed method has been confirmed across many benchmark problems, as shown below. Note that, as already mentioned in Section 2.1.4, since the baseline method for satisfying the task-agnostic setting under limited computational resources is limited to DER, subsequent comparisons are conducted only using DER and ablation studies of the proposed method.

**Table 1:** Parameter configuration.

**Symbol**	**Meaning**	**Value**
*N* ^FIFO^	Size of FIFO buffer	512
*N* ^RS^	Size of RS buffer(s)	512
*B*	Batch size	32
α^ini^	Initial α in DER	1
β^ini^	Initial β in DER	0.5
ρ	Quantile for threshold computation	0.5
λ	Smoothness of data priority	0.5
*q*	Parameter for generalized counter(s)	(1.5, 1)
ζ	Rejection probability	0.2

### Results of A2ER

#### Toy problems

**Figure 6:**
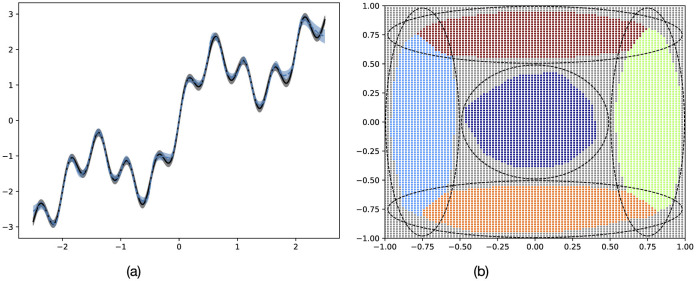
Examples of toy problems. **(a)** Regression. **(b)** Classification.

First, to validate the effectiveness of A2ER, simple regression and classification problems were conducted, as shown in Figure 6. These detailed conditions are described later, but due to factors such as low learning frequency, they were set such that learning cannot be satisfactorily achieved unless the algorithm possesses sufficient plasticity while keeping consolidation as well. A neural network model consisting of two fully connected layers with 32 neurons each was trained for both toy problems. The model outputs a normal distribution for the regression problem and a categorical distribution for the classification problem. In both cases, the model was optimized by minimizing the negative log-likelihood with respect to the supervised data as the loss function.

The regression problem aimed to predict the one-dimensional output of a composite function of up to seven sine waves with different phases, frequencies, and amplitudes, given a one-dimensional input in the range [−2.5, 2.5]. During training, outputs with white noise (SD = 0.1) were obtained as the input increased in 0.001 increments from the minimum value, and the resulting input-output pairs were passed to the model sequentially. One cycle contained 5,000 data points, exceeding the buffer size, and was repeated five times to achieve sufficient accuracy. Training occurred after every 16 data points were added, and up to 16 batches were replayed from each buffer during a single training session. This problem was evaluated using the sum of Kullback-Leibler divergences (KLD) between the predicted and true distributions.

The classification problem aimed to predict which component of a Gaussian mixture distribution, comprising up to 16 components placed on a two-dimensional input space [−1, 1]^2^, a given input belongs to, or whether it belongs to none. During training, the input space was divided into grids at intervals of 0.02, with added white noise (SD = 0.01). The outputs were the indices of the components with the highest likelihood. A threshold of 0.3^2^ was set; if all likelihoods were below this threshold, the input was assigned an additional index, indicating outliers. This cycle was repeated five times for 10,000 data points to ensure accurate classification. Training was performed after every 32 new data points were added, and up to 16 batches were replayed from each buffer in a single training session. The task was evaluated using classification accuracy (ACC).

Using the above setup, four types of functions (sine waves and Gaussian mixture distributions) were prepared. They were trained under the following methods, each statistically evaluated using 20 different random seeds.

- *DER*: the conventional method
*-Aa*: without the *adaptation* strategy for α
*-Ab*: without the *adaptation* strategy for β
*-B*: without the *block* strategy
*-C*: without the *correction* strategy
- *A2ER*: the proposed method

Because the purpose of this section is to evaluate A2ER, the conventional implementation of RS (not O2S) was employed.

The learning results are summarized in Table 2. To evaluate robustness across problems, a weighted average based on the ranks of the evaluation values was used to prioritize the worst cases. The conventional method, DER, was never among the top-2, perhaps because it was not fine-tuned and did not perform sufficiently well. The performance without the *correction* strategy was significantly worse than that of the conventional method. This may be because the *adaptation* strategy assigns too much weight to α without the reduction of the threshold Δ_*Q*_ by the *correction* strategy, inhibiting learning.

**Table 2:** Results of toy problems for evaluating A2ER: the average over 20 trials per condition was weighted by rank, prioritizing to the worst case; top-2 results are in bold.

**Method**	Regression (KLD: ↓)				Classification (ACC: ↑)			
	**R1**	**R2**	**R3**	**R4**	**C1**	**C2**	**C3**	**C4**
DER	0.84	4.00	7.24	2.23	84.12	73.25	80.97	84.58
	0.64–0.92	3.71–4.18	6.81–7.48	1.98–2.32	82.69–85.82	71.37–75.64	77.81–83.91	82.70–86.82
-Aa	0.49	**2.83**	7.72	1.72	89.99	85.82	89.88	92.56
	0.28–0.73	2.29–3.36	4.91–13.36	1.51–1.86	88.83–91.57	85.18–87.45	87.85–92.26	91.67–93.98
-Ab	**0.40**	3.07	**6.59**	**1.63**	90.00	85.91	90.74	**96.45**
	0.26–0.49	2.50–4.35	4.43–8.29	1.44–1.93	88.30–92.21	84.92–87.90	89.61–93.17	95.94–97.19
-B	**0.36**	2.90	7.12	**1.69**	**91.33**	**87.63**	**91.77**	95.79
	0.28–0.45	2.36–3.13	4.15–12.07	1.44–1.85	90.61–92.76	86.71–88.34	90.94–93.40	95.29–96.63
-C	1.56	6.19	10.44	2.14	65.99	39.68	62.24	64.78
	0.83–1.96	5.56–6.41	9.50–10.66	1.81–2.34	63.38–71.11	35.69–47.84	60.25–67.82	59.90–71.34
A2ER	0.42	**2.87**	**7.03**	1.71	**91.26**	**87.52**	**91.28**	**95.82**
	0.30–0.49	2.37–3.59	4.44–10.68	1.39–1.78	90.07–92.70	87.03–88.70	89.89–93.39	95.33–96.85

Bold indicates top results.

Under the other conditions where the *correction* strategy was added, the performances were better than that of the conventional method. In particular, the proposed method, A2ER, achieved the highest number of top-2 entries. However, the performance without the *block* strategy was similar, and its usefulness was not confirmed by these benchmarks. On the other hand, by focusing on the case without the *adaptation* strategy, we can find that the automatic adjustment of α is particularly effective. When β was not auto-tuned, the performance degraded mainly in the classification problems. In fact, while β in A2ER stayed around 0.5 for the regression problems, it temporarily increased to over 0.8 for the classification problems. This means that the automatic adjustment of β improves performance by steering it toward an appropriate value, even when the initial setting is not optimal.

The above results indicate that the *adaptation* and *correction* strategies of the proposed method are necessary to improve the performance of DER. The *block* strategy may be activated only when inconsistent data remain in the buffers because of, for example, shifts in the data-generative distributions. The benchmark problems above do not have such characteristics; thus, the effectiveness of the *block* strategy could not be confirmed.

#### Reinforcement learning

To verify the effectiveness of the *block* strategy, additional benchmarks for reinforcement learning (RL) problems, where the data-generative distribution depends on the agent's policy, were conducted. As data generated with past policies would be inconsistent with the current policy, reverting to the inconsistent outputs might cause drastic policy updates, which are prone to make RL unstable (Schulman et al., 2017; Saglam et al., 2023). If their replay can be appropriately blocked, a policy should be smoothly updated, improving control performance steadily.

Specifically, two problems in OpenAI Gym, *InvertedDoublePendulum-v4* (DoublePendulum) and *Reacher-v4* (Reacher), were solved using the soft actor-critic (SAC) algorithm (Haarnoja et al., 2018). The SAC implementation was adapted from that used in the literature (Kobayashi, 2025). The training occurred after every four interactions, during which up to one batch was replayed for training. DoublePendulum and Reacher were solved with 1,500 and 1,000 episodes, respectively, followed by 100 episodes using the trained policies for evaluation. Note that these tasks were selected as toy problems expected to be solvable even with small buffers, although their performance was lower than when trained with rich computational resources and buffers. In the RL problems, the interquartile mean (IQM) of the 100 episodes' returns (i.e., the sum of rewards in an episode) is computed as the score for each trial, according to the literature (Agarwal et al., 2021).

The following four conditions were compared for the above tasks using 20 random seeds:

- *FIFO*: using only a FIFO buffer, similar to standard RL algorithms with experience replay (Lin, 1992; Isele and Cosgun, 2018).
- *DER*: the conventional method.
*-B*: the proposed method without the *block* strategy.
- *A2ER*: the proposed method.

To ensure fairness, the buffer size in the case using only the FIFO buffer was set to *N*^FIFO^←*N*^FIFO^+*N*^RS^ = 1024, and the batch size was set to *B*×2 = 64. Note that even with an enlarged FIFO buffer, its size remains much smaller than those typically used in general RL implementations, and generalization performance is expected to decrease because it cannot retain sufficient past information. On the other hand, DER and the proposed method, which include RS buffers and regularization to past outputs, can improve generalization performance. However, excessive consolidation of past outputs may disturb or stop learning because some past data become inconsistent or non-optimized because of distribution shifts or insufficient learning.

The learning results are summarized in Table 3. As well as the above benchmarks, a weighted average based on the ranks of the scores was used to prioritize the worst cases and demonstrate robustness. The first notable point is that DER made little progress in learning. This is because the regularization term to maintain past outputs was too strong with α = 1, which prevented the value function from being updated and led to failure in policy optimization. In addition, FIFO, a common implementation in RL, showed a poor success rate on DoublePendulum. In Reacher, on the other hand, FIFO did not fail significantly, although its score did not reach a satisfactory level. This is probably because, although learning proceeded due to high plasticity, the generalization performance to reach arbitrary target positions degraded due to the lack of consolidation caused by the small buffer size.

**Table 3:** Results of RL tasks for evaluating A2ER: IQM of 100 returns was employed as a metric (higher is better); the average over 20 trials per condition was weighted by rank, prioritizing to the worst case.

**Method**	**RL (IQM of returns:** ↑**)**	
	**DoublePendulum**	**Reacher**
FIFO	873.66	-12.29
	206.71–9359.81	-13.05—10.13
DER	60.55	-34.86
	35.53–129.96	-52.19—12.50
-B	1905.71	-16.08
	130.18–9359.86	-27.75—9.72
A2ER	**2896.43**	**-11.69**
	353.61–9359.65	-12.51—8.43

Bold values indicate top result.

However, the proposed method, A2ER, outperformed the other methods on both problems. Although Reacher's score seems not to differ significantly from that of FIFO, both the worst and best cases of A2ER were better than those of FIFO, indicating that A2ER provided more stable generalization performance. This result is largely owing to the *block* strategy, as a clear performance drop was observed in its absence. In particular, the number of failure cases increased for both problems, with the worst case for Reacher being more than twice as severe. Thus, the *block* strategy mitigated the influence of inconsistent past data, which causes instability in learning and deterioration of generalization performance.

Based on the above results, we can conclude that all three strategies in the proposed method, A2ER, are essential for enhancing the performance of DER.

### Results of O2S

Next, O2S, a modified RS, was evaluated using a goal-conditioned RL (Liu et al., 2022) problem, which demand both memory consolidation and plasticity. In such problems, an agent must learn a policy capable of achieving any goal, with different goals randomly assigned in each episode. If the implementation relies solely on a (small) FIFO buffer and is overly plastic, it may quickly forget how to achieve earlier goals conditioned in the first half of the learning process. Conversely, if the system emphasizes consolidation too strongly, such as with the standard RS, it may fail to adapt to goals conditioned in the second half of the learning process. An appropriate balance can be achieved by leveraging the buffers' ability to retain past data, alongside moderate updates with new data and the active exclusion of inconsistent data.

**Figure 7:**
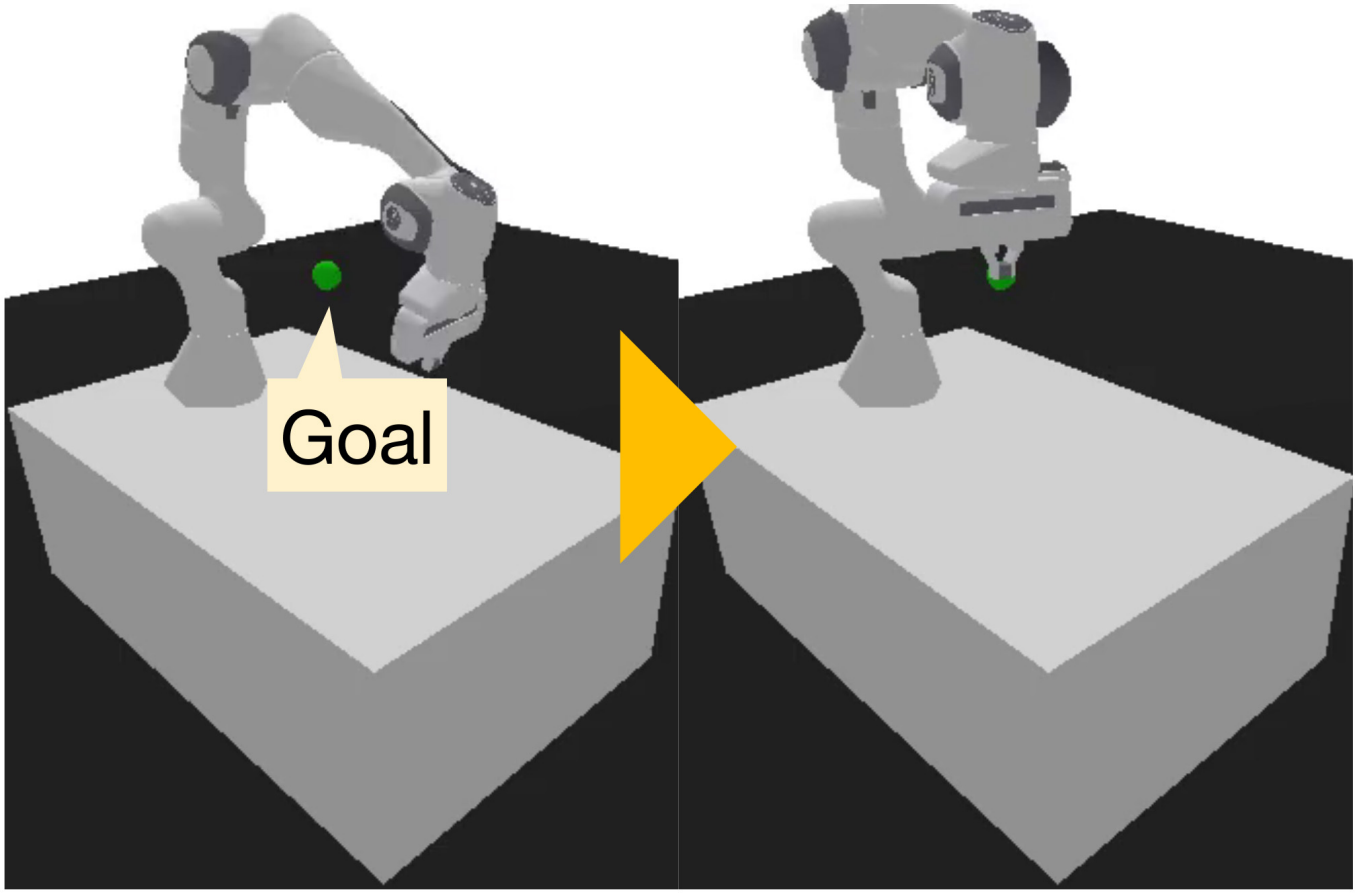
Snapshots of goal-conditioned RL.

This problem is based on a modified version of *PandaReachDense-v3* in Panda-Gym (Gallouédec et al., 2021) (see Figure 7). Three key modifications were introduced. First, the initial joint angles of the robot were randomized using a uniform distribution. Second, the goal position for the robot's end effector was vertically offset by the length of the fingertips, with the offset range specified by a uniform distribution. Third, when the joint space was used as the action space, an orientation error penalty for the fingertip was added to the reward function. In addition, the end-effector orientation and joint angles were appended to the state space (originally, the end-effector position and velocity) to support this modification. Under these modifications, training was conducted for over 10,000 episodes using SAC with A2ER, following the same protocol as in the previous RL problems. The problems were categorized into three types based on the inclusion of noise: uniform noise with a width of π/4 added to the initial joint angles (N), and/or uniform noise with a width of 0.4 added to the goal position (G). Furthermore, the action space was defined either in the 3-DOF end-effector position space (E) or the 7-DOF joint space (J), resulting in six total problem variants.

Because the three strategies in O2S are implemented in stages, performing ablation tests in the same manner as in the previous benchmarks is not feasible. Instead, the strategies were incrementally added to the original RS to assess the individual contributions of each. Accordingly, the following four methods were evaluated:

- *RS*: the conventional method.
- *Q2S*: RS with only the *q-logarithm* strategy.
- *P2S*: without the *omission* strategy.
- *O2S*: the proposed method.

Note that P2S and O2S incorporate the *plural* strategy with *L* = 2 layers; thus, each of the two RS buffers is assigned a size of *N*^RS^/2 = 256.

The results of 20 trials for each condition, using different random seeds, are summarized in Table 4. The evaluation method follows the same procedure used for the RL problems described earlier. A general trend observed was that tasks using the joint space were more challenging than those using the end-effector position space, resulting in lower returns (partly owing to the inclusion of the orientation error penalty). When noise was added only to the initial joint angles, fewer than 10,000 episodes were typically sufficient for learning, as similar states could be encountered through trial-and-error. However, the difficulty increased significantly when noise was added to the goal position. In such cases, the conventional RS failed to learn an optimal policy capable of reaching multiple goals.

**Table 4:** Results of goal-conditioned RL tasks for evaluating O2S: the average over 20 trials per condition was weighted by rank, prioritizing to the worst case; top-2 results are in bold.

**Method**	End-effector space			Joint space		
	**E+N-G**	**E-N+G**	**E+N+G**	**J+N-G**	**J-N+G**	**J+N+G**
RS	-3.62	-6.25	-6.84	-7.30	-15.64	-16.13
	-5.63—1.70	-11.57—3.21	-11.38—3.52	-11.86—4.36	-19.80—8.02	-21.14—11.71
Q2S	-2.47	-3.97	**-4.22**	-6.19	-9.14	**-8.13**
	-3.28—1.17	-7.00—2.40	-5.30—3.22	-8.58—3.83	-12.06—5.19	-9.64—5.81
P2S	**-2.25**	**-3.77**	-4.53	**-5.03**	**-9.00**	-8.17
	-3.04—1.34	-6.15—1.94	-5.30—2.85	-8.06—3.70	-16.95—4.19	-9.65—5.55
O2S	**-2.36**	**-3.36**	**-4.11**	**-5.48**	**-7.64**	**-7.63**
	-2.81—1.27	-4.63—2.30	-5.48—2.83	-8.53—3.26	-9.31—4.34	-9.20—5.24

Bold indicates top results.

By contrast, incorporating the *q-logarithm* strategy to suppress the decay of acceptance probability for new data in the RS buffer enabled more goal variations to be retained. This lead to a notable improvement in performance. Although the overall performance gain from the *plural* strategy appeared minor, a clear enhancement was observed in the best-case results, particularly in cases involving randomized goals. That is, although the *plural* strategy does not fully stabilize learning, it has the potential to enhance the expected performance.

Finally, with the addition of the *omission* strategy, top-2 performance was achieved across all problems. In particular, a substantial improvement was observed when goal positions were randomized. Moreover, the worst-case outcomes improved in nearly every case, indicating enhanced stability in learning. These improvements do not appear to stem from increased plasticity, as seen with the *q-logarithm* and *plural* strategies. As shown in Table 5, the final acceptance probability of the *L*-th RS buffer highlights that the conventional RS lacks plasticity, and that this limitation is mitigated by the *q-logarithm* strategy. The *plural* strategy also contributed to increased plasticity, as evidenced by the higher acceptance of new data in the first RS buffer. However, the increase in acceptance probability resulting from the *omission* strategy was small, indicating that it does not significantly affect plasticity. Instead, this suggests that the *omission* strategy primarily aids in data selection. By actively removing inconsistent data, it maintains a more informative buffer, thereby promoting more stable learning.

**Table 5:** Final acceptance probability of the *L*-th RS buffer.

**Method**	**Condition**	**Probability [%]**
RS	*L* = 1, *q* = 0	0.10
Q2S	*L* = 1, *q* = 1	12.69
P2S	*L* = 2, *q* = 1, ζ = 0	13.32
O2S	*L* = 2, *q* = 1, ζ = 0.2	**13.43**

Bold values indicate top result.

These results indicate that all three strategies incorporated in the proposed method, O2S, are essential for enhancing the performance of RS.

### Image classification benchmarks compared to the state-of-the-art methods

Finally, an in-depth investigation of the proposed method's performance is conducted using common CL benchmarks and other baseline methods, which satisfy the problem settings of the proposed method. The first benchmarks are Permuted MNIST and Split MNIST. Both involve sequentially learning five tasks. To increase problem complexity, Random Erasing (Zhong et al., 2020) with default settings is applied to the training data. To match this increased complexity, the number of neurons in the two fully connected layers is increased to 512, and the batch size is increased to 128. The learning frequency is set to one batch per 128 data points passed. Furthermore, to highlight the importance of plasticity, the learning rate is reduced to 10^−5^, and the number of epochs per task is limited to five. Therefore, the final classification accuracy is considered a reference record, and the focus is on relative accuracy differences.

The comparison methods include the basic DER and the proposed methods, A2ER and O2S (over A2ER), along with two state-of-the-art general-purpose CL methods that incorporate plasticity. Note that for the target benchmarks, memory consolidation is more important than in the above experiments (even with modified conditions emphasizing plasticity), so the O2S hyperparameters were adjusted to (*q*_1_, *q*_2_) = (0.5, 0.0).

Specifically, the first baseline is greedy sample selection (GSS) (Aljundi et al., 2019c). GSS selectively stores highly-novel data compared to the buffer's data, making the model plastic. Although GSS needs expensive per-sample gradients to compute novelty, its settings are enough general as like the proposed method. It is possible to combine DER's function regularization, but the implemented GSS stands on experience replay (i.e., DER with α = 0) to align with the original implementation. Second is continual back propagation (CBP) (Dohare et al., 2024). CBP enhances plasticity by probabilistically reinitializing parameters with low contribution, which is added to the basic DER in this experiment. However, CBP's default settings are optimized for extremely long-term streams, making reinitialization infrequent in this experiment. Therefore, the probability of reinitialization was increased from 10^−4^ to 10^−3^. Furthermore, since the regularization term that retains past outputs in the basic DER obviously interferes with reinitialization, α = 0.1, smaller than the default (i.e., α = 1), is set. Third is layerwise proximal replay (LPR) (Yoo et al., 2024). LPR implicitly retains past (layer-wise) outputs by projecting gradients. Since it updates the projection matrix at fixed intervals (default 10), it maintains plasticity without retaining overly old past outputs like the basic DER. However, adding LPR to the basic DER would have potential loss of the deliberately retained plasticity. Therefore, LPR is combined with experience replay (i.e., DER with α = 0). The hyperparameter ω was set to 1, one of the recommended values. Fourth is gradient-guided epsilon constraint (GEC) (Lai et al., 2025). GEC treats CL as a constrained optimization problem, similar to the proposed method, and allows adjusting plasticity through the tolerance of the constraint. Like others, GEC can also combine DER's function regularization, but the implementation follows the original paper without it. Note that hyperparameters were left as original.

**Figure 8:**
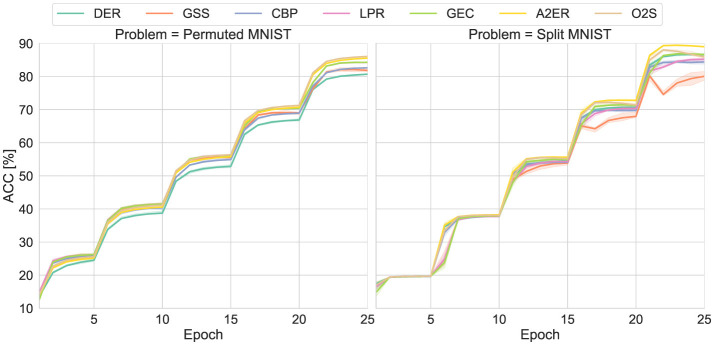
Learning curves of permuted MNIST and split MNIST with five tasks.

Under the above conditions, the learning curves for the test data obtained from 20 trials with different random seeds are plotted in Figure 8. In the basic DER, its low plasticity slowed down learning in both problems, preventing the achievement of sufficient classification accuracy. GSS declined in classification performance on Split MNIST, likely because its buffer was easily overwritten by new class data. In CBP, the classification accuracy for Permuted MNIST was improved from the basic DER. However, probably due to the reset of classifier for past classes (i.e., a kind of intentional forgetting), CBP did not achieve sufficient overall classification accuracy on Split MNIST. Similarly, LPR was able to quickly learn new data while maintaining classification accuracy for past data on Permuted MNIST (though hidden behind the line of GEC), but on Split MNIST, its learning lagged behind the basic DER. This might be because the gradients for learning the new class classifier tends to vanish by the projection, and it took time for the projection adjustment for the new classes. Among the baselines, only GEC achieved classification performance superior to or equivalent to DER for both problems. Furthermore, GEC's computational time and memory usage were almost the same as that of DER and the proposed methods, while the others showed a significant increase.

Compared to these baselines, A2ER obviously achieved superior classification accuracy on both Permuted MNIST and Split MNIST. This demonstrates that the ability to automatically adjust the balance between consolidation and plasticity according to the problem is effective. On the other hand, while adding O2S enabled the highest classification accuracy on Permuted MNIST, it caused catastrophic forgetting in the final task on Split MNIST, leading to a decline in classification accuracy. As mentioned earlier, although the O2S setting prioritized consolidation, it still appears to have resulted in excessive plasticity. Further investigation into automatic hyperparameter tuning and appropriate buffer size allocation might be warranted.

Based on the above results, the more challenging CIFAR-10/100 are solved in a task-incremental setting with 2/10 tasks, respectively. Since this requires image inputs, ResNet18 is employed as the classification model, while its batch normalization was replaced by group normalization. The buffer size was increased to 5120 due to data diversity, but all other conditions are the same as for MNIST.

**Figure 9:**
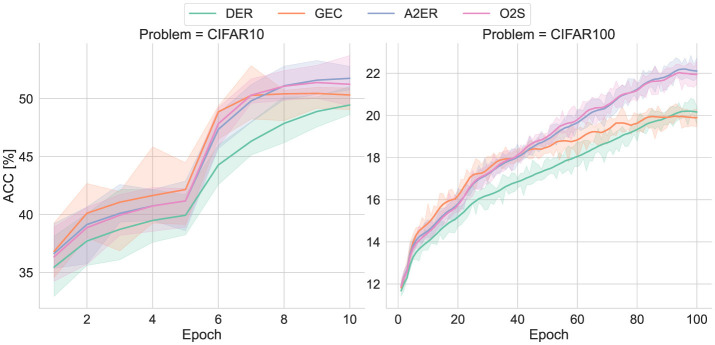
Learning curves of CIFAR-10 with two tasks and CIFAR-100 with 10 tasks.

Referencing the results on MNIST, only GEC was tested as the state-of-the-art method since only it showed high similarity to the proposed method and also delivered superior results (classification performance and computational efficiency). That is, DER, GEC, A2ER, and O2S are compared, as depicted in Figure 9. Note that these learning curves were statistically obtained from 10 trials with different random seeds.

Similar to the MNIST case, DER struggled to progress through training due to insufficient plasticity, resulting in inadequate classification accuracy. GEC efficiently acquired new knowledge and improved classification accuracy early in training, but its insufficient consolidation led to forgetting, preventing sufficient performance gains in later tasks. Both the proposed A2ER and O2S achieved higher classification accuracy than these baseline methods, suggesting they achieved an appropriate balance between consolidation and plasticity. Note that forgetting did not occur with O2S, as it did with MNIST, due to the increased buffer size.

## Conclusion

This paper proposed A2ER and O2S, which introduce three improvement strategies for DER and RS, respectively, as versatile baselines for continual learning without requiring task label information and rich computational resources. Conventional methods primarily focus on maintaining past data and outputs, i.e., memory consolidation, which often compromises memory plasticity, or the ability to adapt to new data, because of the inherent trade-off between the two. The proposed methods were designed to improve this trade-off and achieve a more effective balance between consolidation and plasticity. Numerical experiments demonstrated that each of the proposed strategies contributed to improved performance across benchmarks where both consolidation and plasticity are crucial.

Furthermore, since the proposed A2ER and O2S are designed for operation on systems with limited computational resources, there is no theoretical difference in computational cost compared to the conventional DER, unless the total buffer size and batch size are increased according to *L*>1. In fact, there was little difference in the training time required for each method during the experiments. This lightweight computational cost not only enables operation in small-scale systems like those discussed in this paper but also suggests scalability to large-scale problems.

However, the proposed method involves several hyperparameters, even though they are robust to problems. As future work, automatic tuning of these parameters is an important next step. In this context, it will be essential to assess whether the problem requires greater consolidation or plasticity, and to accurately identify which data should be discarded from replayed or storage. Achieving this would enable the proposed method to be applied for the training of large-scale practical models on extensive data streams. In particular, the proposed method holds promise in domains where data volume increases continuously, such as robotic foundation models (Firoozi et al., 2023) and the analyses of complex human motions (Kong and Fu, 2022). By conducting a comprehensive comparison with recent continual learning methods in these domains, it is believed that the proposed A2ER and O2S will be established as the standard in this field.

## Data Availability

The raw data supporting the conclusions of this article will be made available by the authors, without undue reservation.

## Footnotes

1 Stability is generally used here rather than consolidation, but in this paper, it is avoided to distinguish from other forms of stability, such as control or learning stability.
