# Supplementary material for: Improvements to dark experience replay and reservoir sampling for better balance between consolidation and plasticity
Source: Front Artif Intell. 2026 Feb 19;9:1649239. doi: 10.3389/frai.2026.1649239 (PMC12961966; doi:10.3389/frai.2026.1649239)
Supplement: Supplementary file 1 [file Supplementary_file_1.pdf]

Supplementary Material:
Improvements to Dark Experience Replay and Reservoir Sampling for Better Balance Between Consolidation and Plasticity

Taisuke Kobayashi [1]

[1] *National Institute of Informatics (NII) and The Graduate University for Advanced Studies (SOKENDAI), Tokyo, Japan*

Correspondence*:
Corresponding Author
kobayashi@nii.ac.jp

APPENDIX

1 PSEUDO CODES OF A2ER AND O2S

The pseudo-codes for the proposed A2ER and O2S algorithms are described in Alg. 1 and Alg. 2, respectively. Each time data is acquired, the loop in Alg. 2 is executed to store data in the buffers, then the loop in Alg. 1 is called to perform learning. In addition, their sample codes are uploaded on GitHub `https://github.com/prinlab/A2ER_O2S`.

2 COMPUTATIONAL COST

The proposed methods A2ER and O2S theoretically have the same computational complexity as the baseline DER. However, an increase in computational cost (i.e., time and memory usage) is anticipated due to implementation overhead and the accumulation of minor redundant computations. To assess the extent of this increase, a relative evaluation of computation time and memory usage is conducted using the classification problem as an example. The experimental system configuration was as follows: CPU is two Intel(R) Xeon(R) CPU E5-2660; OS is Ubuntu 22.04.3 LTS; and Python version is 3.10.12.

The ratios between the methods including the ablation tests and DER are summarized in Table 6. Note that the methods were not fully optimized in their implementation; therefore, the ablation tests in particular should be considered reference records. Therefore, only the important A2ER and O2S are emphasized in bold. Anyway, A2ER and O2S increased computational time and memory usage by approximately 5 % each, leading to a total cost increase of approximately 10 %. Since the proposed methods are not affected by the size of the network model, this level of increase is considered less of a bottleneck, the cost increase that occurs when scaling up the model size. Indeed, when the model size was increased from two fully connected layers to ten layers, the ratios of computational time and memory usage were suppressed from 12.9 % and 7.4 % to 10.9 % and 4.9 % increments in O2S, which is with the largest cost originally, respectively.

Algorithm 1 Pseudocode of A2ER

Initialize α^{real}, β^{real}, $\Delta_Q = 0$
while data is passed **do**
  $t+ = 1$
  if $t\%H = 0$ **then**
    for $i = 1$ to E **do**
      Sample data from FIFO buffer uniformly: $B^{\text{FIFO}} = \{(x_\tau, y_\tau)\}^{|B|} \subseteq D^{\text{FIFO}}$
      Sample data from RS buffer with the probability $\propto \bar{\gamma}_\tau$: $B^{\text{RS}} = \{(x_\tau, y_\tau, z_\tau, \bar{\gamma}_\tau)\}^{|B|} \subseteq D^{\text{RS}}$
      Compute $\mathcal{L}^{\text{FIFO}} = \sum_{\tau \in B^{\text{FIFO}}} \mathcal{L}(g(h_\theta(x_\tau)), y_\tau)/|B|$
      Compute $\mathcal{L}^{\text{RS}} = \sum_{\tau \in B^{\text{RS}}} \mathcal{L}(g(h_\theta(x_\tau)), y_\tau)/|B|$
      Compute $\mathcal{L}^{\beta} = -\beta^{\text{real}}\{\text{sg}(\mathcal{L}^{\text{RS}}) - \text{sg}(\mathcal{L}^{\text{FIFO}})\}$
      Compute $z_\tau^{\text{new}} = h_\theta(x_\tau)$ for $\tau \in B^{\text{RS}}$
      Compute $\Delta_\tau = \|z_\tau^{\text{new}} - z_\tau\|_2^2/2$ for $\tau \in B^{\text{RS}}$
      procedure WITH NO GRADIENT COMPUTATION
        Update $\Delta_Q = (1 - |B|/N^{\text{RS}})\Delta_Q + |B|/N^{\text{RS}} Q(\{\Delta_\tau\}_{\tau \in B^{\text{RS}}}; \rho)$
        Compute $\eta_\tau = 1 - \rho\{\text{clip}(\Delta_\tau; \Delta_Q, \Delta_Q/\rho) - \Delta_Q\}/\{(1-\rho)\Delta_Q\}$ for $\tau \in B^{\text{RS}}$
        Compute $\gamma_\tau = 1 - \sqrt{\eta_\tau + (1 - \eta_\tau)\Delta_Q/\Delta_\tau}$ for $\tau \in B^{\text{RS}}$
        Update $\bar{\gamma}_\tau = (1 - \lambda)\bar{\gamma}_\tau + \lambda(1 - \gamma_\tau)$ for $\tau \in B^{\text{RS}}$
        Update $z_\tau = (1 - \gamma_\tau)z_\tau + \gamma_\tau z_\tau^{\text{new}}$ for $\tau \in B^{\text{RS}}$
      end procedure
      Compute $\mathcal{L}^{\text{DER}} = \sum_{\tau \in B^{\text{RS}}} (1 - \gamma_\tau)^2 \Delta_\tau/|B|$
      Compute $\mathcal{L}^{\alpha} = -\alpha^{\text{real}} \sum_{\tau \in B^{\text{RS}}} \eta_\tau(\text{sg}(\Delta_\tau) - \Delta_Q)/|B|$
      Compute $\beta = \text{sigmoid}(\text{sg}(\beta^{\text{real}}))$
      Compute $\alpha = \text{softplus}(\text{sg}(\alpha^{\text{real}}))$
      Comute $\mathcal{L} = (1 - \beta)\mathcal{L}^{\text{FIFO}} + \beta\mathcal{L}^{\text{RS}} + \alpha\mathcal{L}^{\text{DER}} + \mathcal{L}^{\beta} + \mathcal{L}^{\alpha}$
      Minimize $\mathcal{L}$ by stochastic gradient descent
    end for
  end if
end while

Table 6. Computational cost compared to DER when running the classification problem

Metric [%]	Model	-Aa	-Ab	-B	-C	**A2ER**	Q2S	P2S	**O2S**
Time	Normal	103.3	107.7	102.0	105.7	**105.9**	109.4	108.7	**112.9**
Memory usage	Normal	105.0	106.0	103.7	104.8	**105.0**	105.4	106.2	**107.4**
Time	Large	104.9	109.3	103.7	106.1	**108.4**	109.6	108.9	**110.9**
Memory usage	Large	104.8	105.2	102.0	104.4	**105.8**	104.7	104.2	**104.9**

3 INFLUENCE OF QUANTILE OPERATION

The dynamic threshold for the error from previous outputs, required by the *adaptation* strategy in A2ER, is strongly influenced by the quantile parameter $\rho \in [0, 1]$. In addition, ρ also affects the calculation of the correction rate in the *correction* strategy, which affects the *block* strategy. Therefore, ρ play a critical role in all three A2ER strategies.

To evaluate its impact, the toy problems described in Section 3.2 were tested with $\rho = 0.25, 0.5, and 0.75$ (corresponding to the quartile points $Q_1/4$, $Q_2/4$, and $Q_3/4$, respectively). The results, summarized in Table 7, show that $\rho = 0.25$ yielded limited improvement to the baseline DER, while both $\rho = 0.5, 0.75$ achieved clear performance gains. In addition, the performance difference between $\rho = 0.5$ and $\rho = 0.75$

Algorithm 2 Pseudocode of O2S

Initialize $D^{\text{FIFO}} = \emptyset$, $D_l^{\text{RS}} = \emptyset$ $(l = 1, \ldots, L)$
while data is passed **do**
  Get data (x_t, y_t) with $t+ = 1$
  Append data in FIFO buffer: $D^{\text{FIFO}} = D^{\text{FIFO}} \cup (x_t, y_t)$
  if $|D^{\text{FIFO}}| > N^{\text{FIFO}}$ **then**
    Discard the oldest data: $d' = \arg\min_t D^{\text{FIFO}}$
  else
    $d' = \emptyset$
  end if
  if $d' != \emptyset$ **then**
    Append variables: $d' = [d', z = h_\theta(x), \bar{\gamma} = 1]$
    Set $p_0^{\text{rej}} = 0$
    for $l = 1$ to L **do**
      Get $\bar{\gamma}_l^{\max} = \max_{\tau \in D_l^{\text{RS}}} \bar{\gamma}_\tau$ and $\bar{\gamma}_l^{\min} = \min_{\tau \in D_l^{\text{RS}}} \bar{\gamma}_\tau$
      Compute $p_l^{\text{rej}} = (\bar{\gamma}_l^{\max} - \bar{\gamma})^\nu / (\bar{\gamma}_l^{\max} - \bar{\gamma}_l^{\min})^\nu$
      Compute $p^{\text{rej}} = 1 - (1 - p_{l-1}^{\text{rej}})(1 - p_l^{\text{rej}})$
      if $p^{\text{rej}} \leq \epsilon \sim [0, 1]$ **then**
        $n_l + = 1$
        if $n_l \leq N^{\text{RS}}/L$ **then**
          Append data in RS buffer: $D_l^{\text{RS}} = D_l^{\text{RS}} \cup d'$
          $d' = \emptyset$
        else
          Sample $k \sim \mathcal{U}(1, f_{q_l}(n_l))$
          if $k \leq N^{\text{RS}}/L$ **then**
            Replace data in RS buffer: $D_l^{\text{RS}}[k] = d'$ and $d' = d_k$
          else
            $d' = \emptyset$
          end if
        end if
      else
        $d' = \emptyset$
      end if
      if $d' = \emptyset$ **then**
        Break
      end if
    end for
  end if
end while

was relatively minor. These findings suggest that A2ER improves learning performance when ρ is set to a large value. For simplicity and robustness, this paper adopts $\rho = 0.5$ as the default setting.

4 OTHER POSSIBLE DESIGNS OF THE COUNTER

The new generalized counter introduced in eq. (22) is a heuristic design, but alternative formulations are also possible. In this case, $0 \leq \Delta f(n) \leq 1$ must be satisfied. However, another important consideration is whether the counter saturates with $\Delta f(n \to \infty) \to 0$. If the counter saturates, the RS buffer continues to accept new data with a nonzero probability, thereby promoting plasticity. Conversely, if the counter

Table 7. Results of toy problems for evaluating the effect of ρ in the quantile operation: the average over 20 trials per condition was weighted by rank, prioritizing to the worst case; top-2 methods are in bold.

Method	Regression (KLD: ↓)				Classification (ACC: ↑)			
	R1	R2	R3	R4	C1	C2	C3	C4
DER	0.84	4.00	7.24	2.23	84.12	73.25	80.97	84.58
	0.64∼0.92	3.71∼4.18	6.81∼7.48	1.98∼2.32	82.69∼85.82	71.37∼75.64	77.81∼83.91	82.70∼86.82
$\rho = 0.25$	0.56	3.33	9.14	1.83	84.08	70.46	82.82	89.66
	0.33∼0.84	2.41∼4.81	4.52∼12.12	1.54∼2.12	83.16∼86.71	69.00∼75.01	81.48∼87.23	88.35∼92.40
$\rho = 0.5$	**0.42**	**2.87**	**7.03**	**1.71**	**91.26**	**87.52**	**91.28**	**95.82**
	0.30∼0.49	2.37∼3.59	4.44∼10.68	1.39∼1.78	90.07∼92.70	87.03∼88.70	89.89∼93.39	95.33∼96.85
$\rho = 0.75$	**0.36**	**2.93**	**6.49**	**1.68**	**91.29**	**87.66**	**91.14**	**95.49**
	0.24∼0.49	2.29∼3.33	4.20∼9.89	1.45∼1.78	90.67∼92.43	87.09∼89.35	89.82∼93.24	94.52∼96.68

does not saturate, the acceptance probability of new data approaches zero over time, which prioritizes consolidation.

With this in mind, two simple candidates are proposed. The first design changes only the slope when $n > N^{\mathrm{RS}}$.

$$f_q^{\mathrm{lin}}(n) = \min(n, N^{\mathrm{RS}}) + \left\lfloor (1-q)\max(0, n - N^{\mathrm{RS}}) \right\rfloor \tag{24}$$

where $q \in [0, 1)$. In this design, the counter diverges to infinity without saturating for $q < 1$.

The second is a saturating design corresponding to exponential decay RS (Cormode et al., 2009; Osborne et al., 2014).

$$f_q^{\mathrm{exp}}(n) = \min(n, N^{\mathrm{RS}}) + \left\lfloor \frac{N^{\mathrm{RS}}}{q} \left\{ 1 - \exp\left(-\frac{q\max(0, n - N^{\mathrm{RS}})}{N^{\mathrm{RS}}} \right) \right\} \right\rfloor \tag{25}$$

where $q \in (0, 1])$. It is noteworthy that $f_{q\to 0}^{\mathrm{exp}}(n)$ converges to n. This design saturates for $q > 0$, and the acceptance probability converges to 0.5 when $q = 1$, as shown in eq. (22) with $q = 2$.

These designs, including the *q-logarithm* strategy, were compared using the classification problem described in Section 3.2. In this evaluation, the target function (i.e., the arrangement of the Gaussian mixture distribution) was switched between the first and second five cycles to assess the balance between consolidation and plasticity. That is, excessive consolidation inhibited learning in the second half, whereas excessive plasticity caused failure in the first half. Therefore, the classification accuracies in the first and second halves reflect the degrees of consolidation and plasticity, respectively. In addition, conditions and trials in which both accuracies exceeded a certain threshold (in this case, 90%) were judged to have an appropriate balance.

The hyperparameter q for each design was divided into 21 equal intervals, and the results of 20 trials for each condition are summarized in Fig. 10. As in the previous experiment, the statistics represent rank-weighted averages that emphasize the worst-case results. The vertical dashed lines in the graphs on the right indicate the conditions under which an appropriate balance was achieved in all 20 trials. For $f_q^{\mathrm{lin}}(n)$, which lacks saturation, an appropriate balance could not be achieved because of insufficient plasticity. In contrast, $f_q^{\mathrm{exp}}(n)$, which includes saturation, exhibited both high consolidation and plasticity; however,

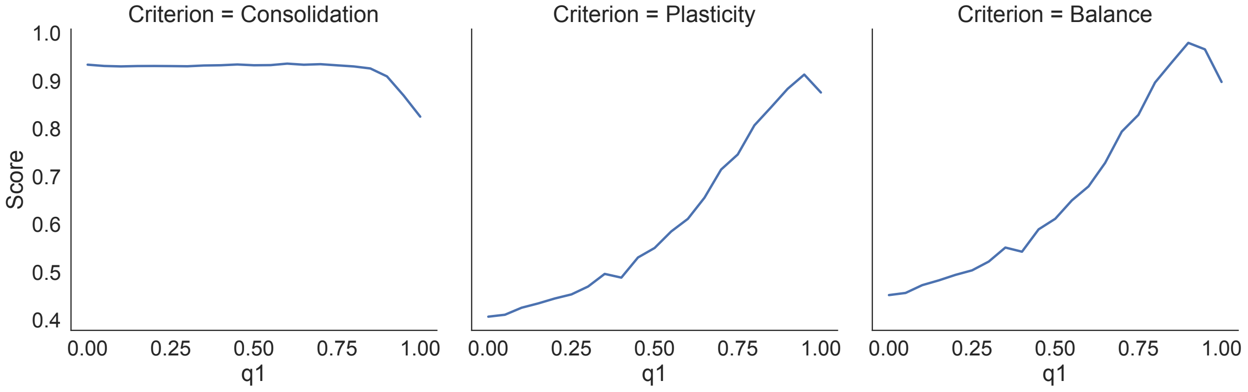

(a) $f_q^{\mathrm{lin}}(n)$

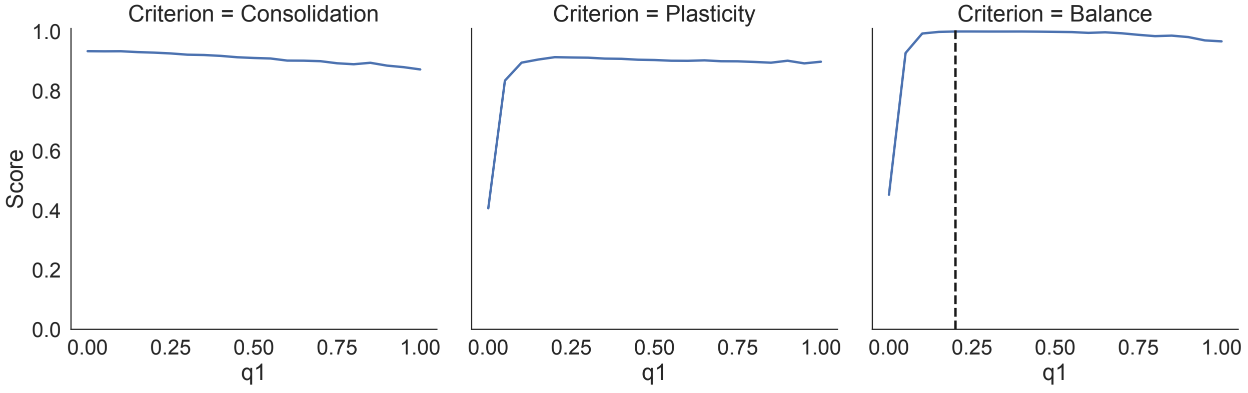

(b) $f_q^{\mathrm{exp}}(n)$

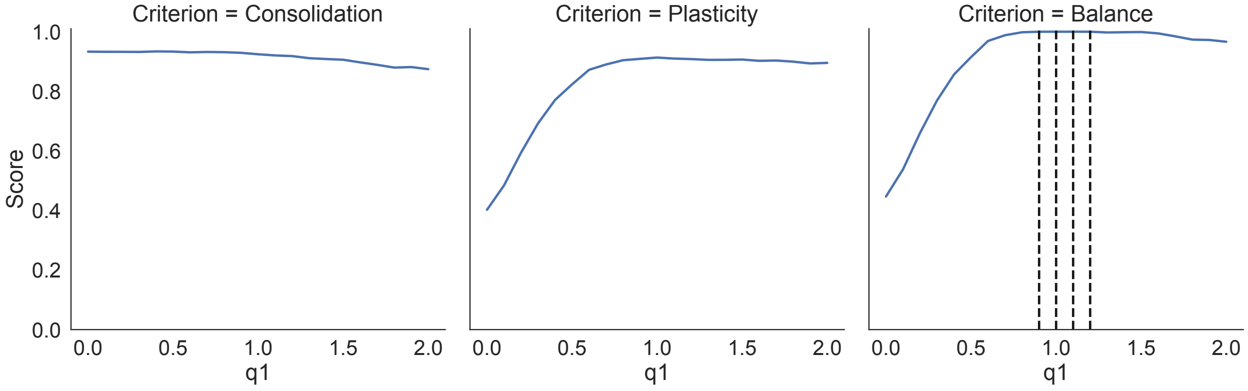

(c) The q-logarithm strategy $f_q(n)$

Figure 10. Comparison of three candidate designs for the generalized counter using the switched classification problem

only one optimal hyperparameter setting was identified. The unexpected high level of consolidation, despite the design's intended lower consolidation, may be attributed to the classification problem not requiring significant consolidation.

By contrast, the proposed *q-logarithm* strategy identified four optimal hyperparameters values at $q = 0.9, 1, 1.1, 1.2$. In particular, $q = 0.9, 1$ correspond to unsaturated behaviour, whereas $q = 1.1, 1.2$ result in saturation, suggesting that an appropriate balance lies near the boundary between saturated and unsaturated regimes. Because the proposed method allows control over saturation through a single hyperparameter, it can robustly achieve a suitable balance between consolidation and plasticity with minimal sensitivity to hyperparameter tuning. Therefore, this design was adopted in this study, with $q = 1$, which is the unsaturated boundary, used as the default. The *plural* strategy allows for saturation in shallower buffers by setting $q > 1$ (e.g., $q = 1.5$ in the first buffer in this study).

REFERENCES

Cormode, G., Shkapenyuk, V., Srivastava, D., and Xu, B. (2009). Forward decay: A practical time decay model for streaming systems. In *IEEE international conference on data engineering* (IEEE), 138–149

Osborne, M., Lall, A., and Van Durme, B. (2014). Exponential reservoir sampling for streaming language models. In *Annual Meeting of the Association for Computational Linguistics*. 687–692
